# Effect of ATorvastatin On Chronic subdural Hematoma (ATOCH): a study protocol for a randomized controlled trial

**DOI:** 10.1186/s13063-015-1045-y

**Published:** 2015-11-18

**Authors:** Rongcai Jiang, Dong Wang, Wai Sang Poon, Yi Cheng Lu, Xin Gang Li, Shi Guang Zhao, Ren Zhi Wang, Chao You, Xian Rui Yuan, Jian Min Zhang, Hua Feng, Zhou Fei, Xin Guang Yu, Yuan Li Zhao, Jin Hu, De Zhi Kang, Ru Tong Yu, Guo Dong Gao, Xi De Zhu, Tao Sun, Jie He Hao, Xian Zhi Liu, Ning Su, Shu Yuan Yue, Jian Ning Zhang

**Affiliations:** Department of Neurosurgery, Tianjin Medical University General Hospital, 154 Anshan Road, Tianjin, 300052 People’s Republic of China; Key Laboratory of Post-trauma Neuro-repair and Regeneration in Central Nervous System, Ministry of Education, 154 Anshan Road, Tianjin, 300052 People’s Republic of China; Tianjin Key Laboratory of Injuries, Variations and Regeneration of Nervous System, 154 Anshan Road, Tianjin, 300052 People’s Republic of China; Tianjin Neurological Institute, 154 Anshan Road, Tianjin, 300052 People’s Republic of China; Oriental Neurosurgical Evidence-based Study Team (ONET) of People’s Republic of China, 154 Anshan Road, Tianjin, 300052 People’s Republic of China; Division of Neurosurgery, Department of Surgery, Prince of Wales Hospital, Chinese University of Hong Kong, Shatin, New Territories East Hong Kong; Department of Neurosurgery, Shanghai Changzheng Hospital, 415 Fengyang Street, Shanghai, 200003 People’s Republic of China; Department of Neurosurgery, Qilu Hospital of Shandong University, 107 Wenhuaxi Street, Jinan, Shandong Province 250012 People’s Republic of China; Department of Neurosurgery, The First Affiliated hospital of Harbin Medical University, 23 Youzheng Street, Nangang district, Harbin, Heilongjiang Province 150001 People’s Republic of China; Department of Neurosurgery, Peking Union Medical College Hospital, 41 Damucang Street, Xicheng district, Beijing, 100032 People’s Republic of China; Department of Neurosurgery, West China Hospital Sichuan University, 37 Guoxuegang Street, Wuhou district, Chengdu, Sichuan Province 610041 People’s Republic of China; Department of Neurosurgery, Xiangya Hospital Central South University, 87 Xiangya Street, Changsha, Hunan Province 410008 People’s Republic of China; Department of Neurosurgery, The Second Affiliated Hospital Zhejiang University School of Medicine, 88 Jiefang Road, Hangzhou, Zhejiang Province 310009 People’s Republic of China; Department of Neurosurgery, Southwest Hospital, 30 Gaotanyanzheng Road, Shapingba district, Chongqing, Sichuan Province 400038 People’s Republic of China; Department of Neurosurgery, Xijing Hospital, 15 Changlexi Road, Xian, Shanxi Province 710032 People’s Republic of China; Department of Neurosurgery, The General Hospital of Chinese People’s Liberation Army, 28 Fuxing Road, Beijing, 100853 People’s Republic of China; Department of Neurosurgery, Beijing Tian Tan Hospital, Capital Medical University, 6 Tiantan Xili, Dongcheng district, Beijing, 100050 People’s Republic of China; Department of Neurosurgery, Huashan Hospital Fudan University, 12 Wulumuqizhong Street, Shanghai, 200040 People’s Republic of China; Department of Neurosurgery, First Affiliated Hospital of Fujian Medical University, 20 Chazhong Road, Fuzhou, Fujian Province 350005 People’s Republic of China; Department of Neurosurgery, The Affiliated Hospital of Xuzhou Medical College, 99 Huaihaixi Road, Xuzhou, Huhehot, Jiangsu Province 221006 People’s Republic of China; Department of Neurosurgery, Tangdu Hospital, The Second Affiliated hospital of the Fourth Military Medical University, 1 Xinsi Road, Xian, Shanxi Province 710038 People’s Republic of China; Department of Neurosurgery, Linyi People’s Hospital, 27 Jiefang Road, Linyi, Shandong Province 276003 People’s Republic of China; Department of Neurosurgery, General Hospital of Ningxia Medical University, 804 Shenglinan Road, Xingqing district, Yinchuan, Ningxia Province 750004 People’s Republic of China; Department of Neurosurgery, First Affiliated Hospital of Shanxi Medical University, 85 Jiefangnan Road, Taiyuan, Shanxi Province 030001 People’s Republic of China; Department of Neurosurgery, The First Affiliated Hospital of Zhengzhou University, 1 Jianshedong Road, Zhengzhou, Henan Province 450052 People’s Republic of China; Department of Neurosurgery, Inner Mongolia people’s Hospital, 26 Zhaowuda Road, Saihan district, Huhehot, Inner Mongolia Province 010017 People’s Republic of China

**Keywords:** Chronic, Subdural hematoma, Atorvastatin, Conservative therapy, Absorption

## Abstract

**Background:**

Chronic subdural hematoma (CSDH) is a common disease that is more prevalent in older people. Surgical intervention is a safe treatment of choice. However, the recurrence rate is relatively high and the outcome is not always satisfactory among surgically treated patients. It is believed that aberrant angiogenesis and intracapsular inflammation contribute to the development of CSDH. Atorvastatin is reported to promote angiogenesis and suppress inflammation. We have recently shown that atorvastatin is effective to non-surgically reduce and eliminate CSDH with minimal side effects. Here, we report a clinical research trial protocol that is designed to evaluate the therapeutic effects of atorvastatin on CSDH.

**Methods/Design:**

We have designed a multi-center, randomized, placebo-controlled, double blind clinical trial for evaluating the efficacy of oral atorvastatin in reducing CSDH. We have so far recruited 96 patients with CT-confirmed or MRI-confirmed CSDHs from 16 medical centers in China. These patients were originally recruited for the Oriental Neurosurgical Evidence-based Study Team (ONET) study. After informed consent is provided, patients are randomized to receive either atorvastatin (oral 20 mg/night for 8 weeks) or placebo (dextrin for 8 weeks); and followed for 16 weeks after the treatment. The primary outcome is the change in hematoma volume at the end of 8-week treatment. Secondary outcomes include: changes in 1) the hematoma volume at the 4^th^, 12^th^, and 24^th^ weeks; 2) Markwalder’s Grading Scale and Glasgow Coma Scale (MGS-GCS); 3) Glasgow Outcome Score (GOS) and 4) Activities of Daily Life – the Barthel Index scale (ADL-BI). Safety will be assessed during the study by monitoring adverse events, laboratory tests, electrocardiography (ECG), measurements of vital signs (temperature, pulse, and blood pressure) and body weight.

**Discussion:**

Results of this trial will provide critical information regarding whether atorvastatin is an effective and safe alternative to surgical treatment of CSDH.

**Trial registration:**

ClinicalTrials.gov Identifier – NCT02024373

The date of trial registration: 7 August 2013

**Electronic supplementary material:**

The online version of this article (doi:10.1186/s13063-015-1045-y) contains supplementary material, which is available to authorized users.

## Background

Chronic subdural hematoma (CSDH) is a common type of encephalic hematoma with an incidence of 5/10,000 in the general population and 58/10,000 in people who are 70 years or older [1]. Head trauma is the most common risk factor for CSDH [2], along with age. CSDH occurs in up to 26.5 % of patients who are 65 years or older. Older patients also have a significantly higher recurrence rate as compared to the general population [3]. Incidences of CSDH and recurrent CSDH are expected to raise worldwide with an aging population. In addition, the increasing use of anticoagulant/antiplatelet therapies is also a common risk factor for CSDH in patients without a history of trauma [4] and poses a challenge for the surgical treatment of CSDH. Unlike subacute subdural hematoma (SSDH), spontaneous reabsorption is rare in CSDH and surgery has traditionally been the treatment of choice [5]. However, the rate of recurrent CSDH is high (approximately 25 %) in surgically treated patients [6], leading to a mortality of 11.1–13.5 % [7]. The surgical outcome is particularly poor for patients of 90 years or older [8].

Because of unsatisfactory outcomes of surgery, several adjuvant or conservative therapies have been clinically tested, including corticosteroids, as complementary therapies [1]. Dexamethasone is reported to effectively reduce the volume of CSDH after surgery [9–12]. It is also reported that perindopril (angiotensin converting enzyme inhibitor, ACEI) reduces the recrudescence of CSDH after surgery [13]. We have recently reported the effect of atorvastatin on reducing CSDH in 23 patients [14].

The pathogenesis of CSDH and mechanisms of above adjuvant therapies remain poorly understood. It has been reported that a CSDH hematoma contains a large number of erythrocytes from continuous blood leakage due to primitively formed blood vessels in the hematoma wall. Vascular endothelial growth factor (VEGF) is detected at a significantly high level in hematoma and in the serum of CSDH patients. [15]. VEGF promotes angiogenesis, but, at a persistent high level, also inhibits the maturation of new vessels [16]. We have previously demonstrated the presence of a pseudo envelope around a subdural hematoma that contains immature and abnormal vessels [17]. In addition, a hematoma is also enriched in proinflammatory cytokines [18–20]. We and other have shown that statins (hydroxy-methylglutaryl-CoA reductase inhibitors) mobilize circulating endothelial progenitor cells (EPCs) to enhance angiogenesis [21–25]. Atorvastatin also inhibits VEGF and reduces the inflammatory reaction [26, 27] as well as up-regulating Notch1/Jagged1 signal, which is critical for VEGF-induced vessel formation and maturation. These reports led us to hypothesize that atorvastatin modulates VEGF expression and the level of inflammation in hematoma, resulting in the shrinkage of CSDH. This protocol was designed to test this hypothesis.

## Methods/Design

### Study objective

The primary objective of this trial is to evaluate the efficacy of atorvastatin in reducing or eliminating CSDHs after 2 months without surgery.

The secondary objectives are to evaluate changes in hematoma volume (non-enhanced computerized tomography (CT) scan) at the 4^th^ week during the treatment; and at the 12^th^ and 24^th^ weeks during the follow-up period; and to measure the effect of atorvastatin on improving neurological function in patients with CSDH as assayed by the MGS-GCS, GOS, and ADL-BI at 4 weeks, 8 weeks and 6 months after atorvastatin therapy is initiated.

### Trial design

The trial is designed as a multi-center, phase II, prospective, double blind, randomized, placebo-controlled study. Eligible patients are randomized in a 1:1 allocation ratio to one of the two treatment arms: 20 mg daily of atorvastatin (treatment) and placebo drug (control). The treatment will continue for 8 weeks followed by 16 weeks of follow-up.

Patients will be recruited from 16 neurosurgery centers in China that are participants in the Oriental Neurosurgical Evidence-based Study Team (ONET) (Table 1, additional ethics documentations in Additional file 1). ONET is chaired by the Department of Neurosurgery, Tianjin Medical University General Hospital. Before the enrollment, patients or their family guardians will be fully informed about the trial, its potential outcomes and adverse events; and provide informed consent. The informed consent forms are in Chinese and English. The study will be conducted according to the Declaration of Helsinki. The ethics boards of Tianjin Medical University General Hospital and other participating hospitals have approved this study.

**Table 1:** Medical centers in China participating in the study

Medical centers	Ethics committee
1. Tianjin Medical University, General Hospital	Medical Ethics Committee of Tianjin Medical University General Hospital
2. Peking Union Medical College Hospital	Ethics Committee of Peking Union Medical College Hospital Affiliated to Chinese Academy of Medical Sciences
3. Prince of Wales Hospital, Hong Kong	Pharmacy and Poisons Board of Hong Kong
4. Qilu Hospital of Shandong University	Medical Ethics Committee of Qilu Hospital Affiliated to Shandong University
5. The First Affiliated Hospital of Zhengzhou University	Clinical Trial Ethics Committee of First Affiliated Hospital of Zhengzhou University
6. The Second Affiliated Hospital of Zhejiang University School of Medicine	Human Research Ethics Committee of the Second Affiliated Hospital of Zhejiang University School of Medicine
7. First Affiliated Hospital of Fujian Medical University	Medical Ethics Committee of First Affiliated Hospital of Fujian Medical University
8. First Affiliated Hospital of Shanxi Medical University	Science/Medical Experimental Ethics Committee of First Affiliated Hospital of Shanxi Medical University
9. General Hospital of Ningxia Medical University	Scientific Experiment Ethics Committee of Ningxia Medical University General hospital
10. Linyi People’s Hospital	Medical Ethics Committee of Linyi People’s Hospital
11. Southwest Hospital The First Affiliated Hospital of the Third Military Medical University	Ethics Committee of the First Affiliated Hospital of Third Military Medical University, PLA
12. Tangdu Hospital, The Second Affiliated Hospital of the Fourth Military Medical University	IEC of Institution for National Drug Clinical Trials, Tangdu Hospital, Fourth Military Medical University
13. The Affiliated Hospital of Xuzhou Medical College	Medical Ethics Committee of Affiliated Hospital of Xuzhou Medical College
14. The First Affiliated Hospital of Harbin Medical University	Medical Ethics Committee of First Hospital Affiliated to Harbin Medical University
15. Xijing Hospital, The First Affiliated Hospital of the Fourth Military Medical University	IEC of First Affiliated Hospital of Fourth Military Medical University
16. Inner Mongolia People's Hospital	Ethics Committee of Inner Mongolia People's Hospital

### Inclusion criteria

The inclusion criteria are CSDH patients who: (1) are ≥ 18 and < 90 years old of either gender; (2) have evidence of supratentorial, unilateral or bilateral CSDH by CT scan (magnetic resonance imaging (MRI) scan is warranted if diagnosis is difficult); (3) have MGS-GCS < Grade 3 (Additional file 2); (4) have no cerebral herniation and immediate needs for surgery (as determined by 2 attending physicians); (5) have had no previous surgery on CSDH; and (6) fully understand the nature of this trial and provide informed consent.

### Exclusion criteria

The exclusion criteria are: (1) allergic to statins or their ingredients; (2) cerebral herniation; (3) subdural hematoma caused by tumors, hematologic diseases or other known severe comorbidities such as multiple organ failure, uncontrolled diabetes mellitus and heart failure; (4) abnormal liver function or liver diseases including uncontrolled hepatitis; (5) other diseases that may interfere with the study as determined by 2 attending neurosurgeons; (6) patients on oral statin medication for more than 1 week before randomization or who are expected to take statins in the next 6 months; (7) patients on oral steroids for more than 1 week before randomization or who are expected to take such medications in the next 6 months; (8) a participant in clinical trials in the past 4 weeks; (9) pregnancy or breastfeeding; (10) failure to complete the trial by poor compliance; (11) other conditions that render a patient unsuitable for the trial as determined by the study investigators.

### Medication and placebo

The 10-mg tablet of atorvastatin is provided by Pfizer, Inc. and taken orally at 20 mg daily before bed. The placebo drug matching oral atorvastatin is made of dextrin with the same weight and appearance and made by Shandong ARURA Pharmaceutical Research & Development. Co., Ltd. with the certification of good manufacturing practice (GMP) for pharmaceutical products issued by the China Food and Drug Administration (CFDA).

Atorvastatin and placebo are packed identically and labeled with packaging number, verification code, dosage, specifications, storage, batch number, the duration of usage, and manufacturer. The package will be labeled: “for clinical study use only.”

### Randomization

Eligible subjects are assigned randomly to either statin or placebo group through the Central Randomization System. The Data Acquisition System for Electronic Data Capture (DAS for EDC) 5.0 (Beijing Stemexcel Technology Co., Ltd., Beijing, China) will be used to produce random numbers. An EDC system is a computerized system designed for the collection of clinical data in electronic format for use mainly in human clinical trials. The ratio of experimental and control group is calculated, by a biostatistician independent of the study data management and statistical analysis, to be 1:1.

After initial screening, an investigator will log onto the Central Randomization System to enter demographic information of an enrolled patient to produce a random number application. A designated individual will then apply for a drug package number and dispense the drug when the package number is consistent with the one recorded in the database.

### Treatment and intervention

*Atorvastatin:* oral intake of a 20 mg Atorvastatin tablet daily for 8 weeks.

*Placebo:* oral intake of a placebo tablet daily for 8 weeks.

At the end of the 8-week treatment, the volume of hematoma will be evaluated by CT scan for the following outcomes:

1. A hematoma disappears or is significantly reduced from the size measured before the treatment. Regardless of whether a smaller hematoma remains, the patient will enter into the follow-up period and should not be continuously treated with atorvastatin.
2. A hematoma does not change significantly or increases by CT scan at the end of 8 weeks of treatment (this can occur in patients who receive placebo). At the end of atorvastatin treatment, attending surgeons can decide if these patients need to receive surgery. The detailed medication and treatment during the process will be continuously recorded for 24 weeks.
3. For any patients whose hematoma increases as determined by CT scan and/or neurological deficits progress during the follow-up period, he or she may undergo surgery. The detailed medication and treatments during the process will be recorded for 24 weeks.

### Timeline schedule

**Fig. 1:**
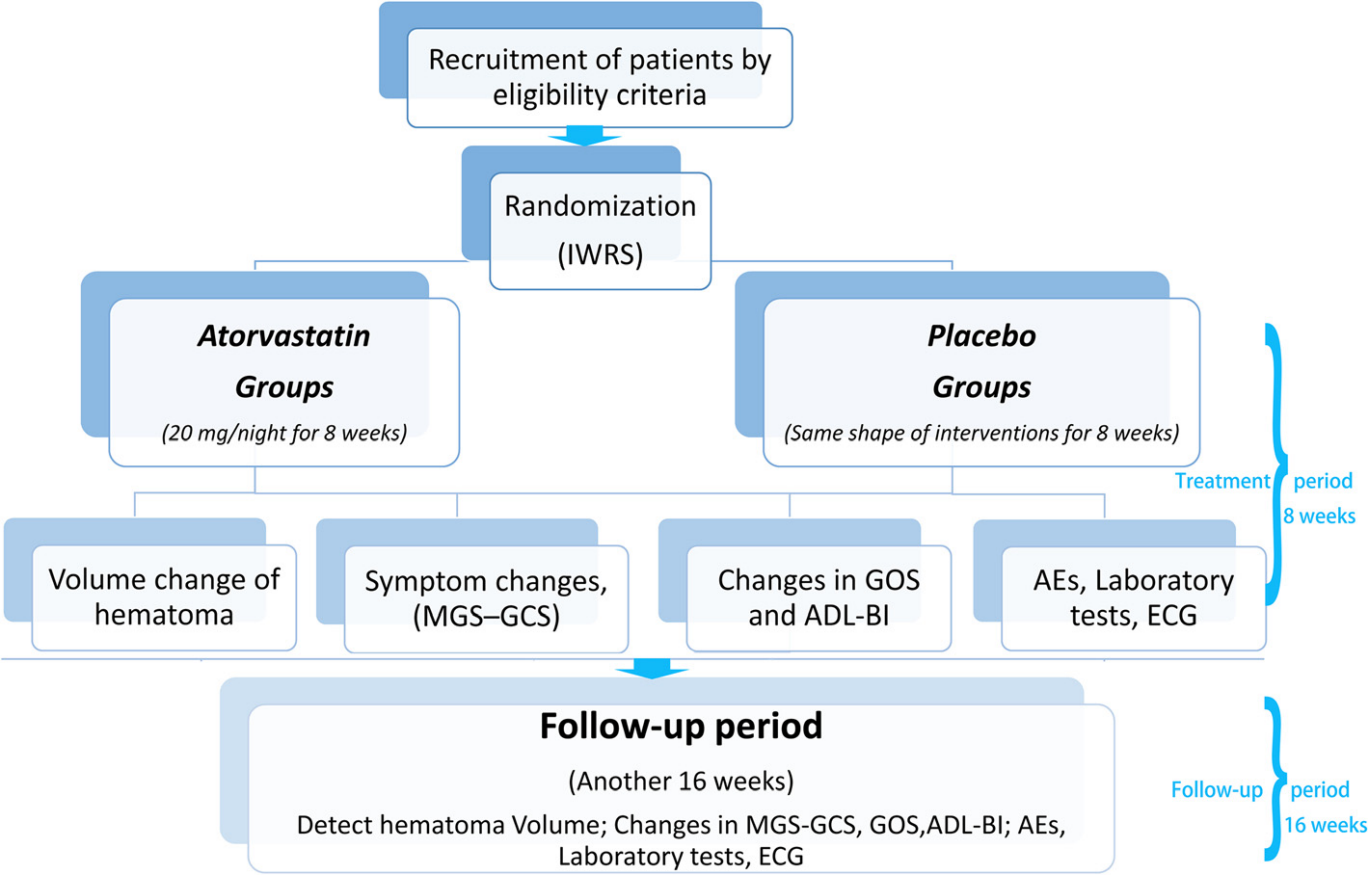
Flow chart to illustrate the design of the trial. *ADL-BI*, Activities of Daily Life – the Barthel Index scale, *AEs* adverse events, *ECG* electrocardiography, *GOS* Glasgow Outcome Score, *IWRS* Interactive Web Response System, *MGS-GCS* Markwalder’s Grading Scale and Glasgow Coma Scale

Protocol-mandated assessments will be performed according to the schedule (Table 2). The study flow chart illustrates key steps of the trial (Fig. 1).

**Table 2:** Visit and assessment schedule

Activities	Screening	Day 1	Days 2–6	Day 7	Week 2 ± 1D	Week 3 ± 1D	Week 4 ± 1D	Week 8 ± 3D (treatment end)	Week 12 ± 3D^4^	Week 24 ± 7D^4^
Informed consent	√									
History collection										
Inclusion/Exclusion	√									
Concomitant medication^1^		√	√	√	√	√	√	√	√	√
Physical examination	√			√	√	√	√	√	√	√
Surgery conversion		√	√	√	√	√	√	√	√	√
Efficiency observation										
Neurological symptoms^2^	√	√	√	√	√	√	√	√	√	√
Hematoma volume by CT^2^	√						√	√	√	√
MGS-GCS/ADL-BI^2^	√			√			√	√	√	√
GOS							√	√	√	√
Safety assessment										
Vital signs	√	√	√	√	√	√	√	√	√	√
Blood routine/Coagulation	√						√	√	√	√
Liver and renal function/Electrolyte/Lipids	√						√	√	√	√
Urinalysis	√							√		√
Electrocardiography	√							√		√
Pregnancy test	√									
Adverse events		√	√	√	√	√	√	√	√	√
Others										
Drug dispensing	√			√	√	√	√			
Drug recovery^3^				√	√	√	√	√		

*ADL-BI* Activities of Daily Life – the Barthel Index scale, *CT* computerized tomography, *D* day, *GOS* Glasgow Outcome Score, *MGS-GCS* Markwalder’s Grading Scale and Glasgow Coma Scale

^1^Records of combination therapy are required until the end of the trial

^2^Surgical therapy is considered in the case of deterioration in neurological symptoms and/or signs, MGS-GCS grading and increase of hematoma immediately imaging review then

^3^The remaining drugs need to be recovered

^4^At weeks 12 and 24, CT should be performed; other laboratory examinations will be selected when needed

### Concomitant care

All patients in the study will receive routine laboratory assessments, including complete blood count (CBC), coagulation (international normalized ratio, INR), liver and renal function, blood lipids and urinalysis. Head CT images will be recorded in Digital Imaging and Communication in Medicine (DICOM) format and evaluated centrally in a triple-blinded manner. Blood samples will be analyzed in a local laboratory and saved in a Case Report Form (CRF) format. The 4 regular follow-up visits are scheduled at the 4^th^, 8^th^, 12^th^ and 24^th^ weeks after the initiation of atorvastatin treatment.

The ACEIs (such as perindopril, captopril, enalapril) are reported to eliminate CSDH [9, 28] and are thus their use is not allowed during the course of atorvastatin treatment. Anticoagulants (warfarin or new oral anticoagulants), antiplatelet drugs (aspirin, clopidogrel) or *Xuesaitong* (traditional Chinese medicine for activating blood circulation) can potentially interfere with hematoma absorption and are not recommended during the treatment. However, these patients will not be exclusively excluded from the trial participation. The following drugs may potentially interact with statins and/or cause adverse events [29] and will not be recommended during the treatment: red yeast rice, erythromycin, fibrates, cyclosporin, macrolide antibacterials, azole antifungals, tacrolimus, gemfibrozil, troglitazone, and itraconazole. The use of any medications will be recorded if they are used concomitantly during atorvastatin treatment in CRF, including name, dosing and duration of these medications.

Most participants are treated as outpatients receiving conventional and standard cares such as rest and pain relief. Family members are responsible for monitoring patients for progress in neurological symptoms such as worsening headache. If a patient is reported to have worsening symptoms, he or she will be further evaluated by two attending physicians for neurological deficits. These patients may be admitted during the trial for treatment of progressing neurological deficits and discharged when their symptoms are relieved significantly. Whether these patients remain on the trial as inpatients will be determined on a case-by-case basis by two participating investigators/surgeons.

### Outcomes

The primary outcome is the change of hematoma volume 8 weeks after atorvastatin treatment on non-enhanced CT scan.

Secondary outcomes are changes of hematoma volume at the 4^th^, 12^th^, and 24^th^ weeks; MGS- GCS, GOS and ADL-BI scores; and improvements in neurological symptoms and signs and laboratory tests.

### Sample size

We have previous shown, in a pilot study of a small patient cohort, that a significant reduction in hematoma volume was found in 95.7 % of CSDH patients treated with atorvastatin. This result provides a base for the power calculation of this trial where we assume that 80 % of patients in the treatment group achieve reduction in hematoma volume and neurological deficits as compared to 50 % reduction in placebo control patients. When the test power (1 − β) is 80 %, we will need to recruit 52 patients in each treatment group based on a 2-size data comparison to reach statistical significance. When a 20 % drop-out rate is taken into the consideration, the sample size is expanded to 126 patients. However, we propose to recruit 200 patients, with 50 % randomly assigned to the atorvastatin treatment group, to meet the requirement of the CFDA for drug registration that the number of cases in treatment group shall not be less than 100 in a phase II clinical trial.

### Data analysis

A 2-tailed test will be performed and a *p* value of less than or equal to 0.05 is considered to be statistically significant. Statistical analysis will be performed by SAS 9.3 (SAS Institute Inc., Cary, NC, USA). The quantitative parameters include group means, standard deviation, median, minimum value and maximum value. A secondary descriptive analysis will summarize improvements of neurological symptoms. For the secondary outcome measures, neurological symptoms and clinical signs of patients in each group are first to be described. Both MGS-GCS and ADL-BI as measured at admission, 3 and 6 months are statistically compared using analysis of covariance (ANCOVA) with the treatment group as a fixed effect and using MGS-GCS and ADL-BI baseline values as covariates.

### Data monitoring

Data monitoring and management will be performed by Stemexcel Technology Company (Beijing Stemexcel Technology Co., Ltd., Beijing, China).

The trial data are recorded in the electronic data management system (DAS for EDC). The CRF is designed according to the protocol in order to collect trial data and to define trial procedures. The form is finalized after sponsors’ approval. CRF data from each participant will be examined and verified by two investigators at a given study site. The entered data will be systematically checked by medically trained personnel. Errors will be corrected by medically trained personnel and communicated to the site investigators. The CRF will be inspected by an assigned person at regular intervals throughout the trial in order to verify the protocol compliance. The entered data should be complete, consistent and accurate.

### SAEs

Severe adverse drug reaction/events (SAEs) refer to the following conditions: death, life-threatening or permanent or significant disability, permanent functional injury to the organs, hospitalization for emergencies or prolonged hospitalization.

Once a SAE is confirmed in a patient, this patient will be removed from the trial and a report should be filled within 24 hours. A full report regarding SAEs should be filled to the sponsor and SFDA. The ethics committee of pharmaceutical affairs of ONET will conduct an investigation to identify causes of SAEs and decide whether the trial will be terminated. The severity of SAE, its relationship with the trial medication, and treatments should be recorded in the CRF. The above conditions are delivered to the ethics committee of all participating hospitals.

### Interim analysis

Interim analysis will not be conducted.

### Auditing

Before the trial is ended, a serial quality control audit of the database will be conducted.

### Methodology

The ATOCH study is designed based on our previous experience in treating CSDH with atorvastatin, showing that 22 out of 23 patients achieved complete hematoma elimination during the follow-up period after atorvastatin treatment. We, therefore, expect a significant volume reduction in the hematomas of patients on atorvastatin. However, since this is a conservative treatment trial of a clinical condition that is traditionally treated surgically, we limit our recruitment to patients who have a small-medium volume of CSDH and whose MGS-GCS is less than 3. To reduce adverse risks, especially patients on placebo, all patients will be closely monitored for acute neurological deterioration. If neurological symptoms deteriorate during the trial, patients will receive an emergency head CT scanning and necessary surgery. Such patients will be removed from the trial.

Except for rhabdomyolysis and impact on liver function, atorvastatin is considered to be generally very safe. Thus, we do not plan to waive SAE for this trial protocol. If a patient develops any suspected related side effects, he or she will be immediately excluded. If atorvastatin-related side effects are confirmed, treatment will be terminated and affected patients will be followed for 6 months.

## Discussion

ATOCH is an atorvastatin-based clinical trial for CSDH. To our knowledge, it is the first complete conservative treatment trial for CSDH, undertaken in order to exclude the influence of surgery as compared to previous trials focusing on reducing the relapse of CSDH after surgery. This trial is based on our previous data [14] and on known effects of statins on modulating inflammation and enhancing angiogenesis [30, 31] as reported for chronic obstructive pneumonic disease (COPD) [32] and acute respiratory distress syndrome (ARDS) [33]. We propose to test atorvastatin, but not other statins, based on our published report on treating CSDH with atorvastatin.

We believe that this trial is necessary because options for conservative treatment of CSDH are very limited [34–36] . While cortisones were found effective in some reports [1, 9–11, 37–39] by primarily blocking inflammation and regulating angiogenesis, they often result in a high incidence of complications at high dose. These complications include digestive tract ulceration, increase in plasma glucose, and deterioration in diabetes [40]. The long-term application of dexamethasone also induces osteoporosis and decreases insulin sensitivity [41]. Perindopril is reported to reduce the relapse rate of CSDH after surgery, but it has not been reported to treat CSDH alone [13]. In contrast, we have shown that atorvastatin is an effective alternative to surgery for CSDH because of its high efficacy and minimal side effects [14]. The most frequent side effect of atorvastatin is myopathy, which is believed to be dose-dependent and spontaneously resolves when atorvastatin is discontinued [42]. The impairment of liver function by atorvastatin is now believed to be very limited [43, 44]. Thus, atorvastatin is expected to be safer than currently available conservative therapies for CSDH. If atorvastatin is proven to be effective through the ATOCH trial, we believe that it can become the therapeutic choice complementary to traditional surgical therapy with or without medical intervention.

## Trial status

The trial started in February 2014 and 96 patients have now been enrolled.

## Additional files

Additional file 1:
**Clinical Trial Certificate of hospital centers participating in the trial.** Medical centers in China participating in the study, ethics committee and the certificate number. (PDF 48 kb) Additional file 2:
**MGS-GCS. **Patients were evaluated using the Glasgow Coma Scale and Markwalder’s Grading Scale. Only patients with Grade 0–2 chronic subdural hematoma (CSDH) were selected for atorvastatin treatment in this study. (PDF 34 kb)

## Abbreviations

ACEI: angiotensin converting enzyme inhibitor
ADL-BI: Daily Life – the Barthel Index scale
ANCOVA: analysis of covariance
ARDS: acute respiratory distress syndrome
ATOCH: ATorvastatin On Chronic subdural Hematoma
CBC: complete blood count
CFDA: China Food and Drug Administration
COPD: chronic obstructive pneumonic disease
CRF: Case Report Form
CSDH: chronic subdural hematoma
CT: computerized tomography
DAS: Data Acquisition System
DICOM: Digital Imaging and Communication in Medicine
ECG: electrocardiography
EDC: Electronic Data Capture
EPCs: endothelial progenitor cells
GMP: good manufacturing practice
GOS: Glasgow Outcome Score
INR: international normalized ratio
MGS-GCS: Markwalder’s Grading Scale and Glasgow Coma Scale
MRI: magnetic resonance imaging
ONET: Oriental Neurosurgical Evidence-based Study Team
SAEs: severe adverse drug reaction/events
SSDH: subacute subdural hematoma
VEGF: vascular endothelial growth factor

## References

[1] Emich S (2014). The efficacy of dexamethasone on reduction in the reoperation rate of chronic subdural hematoma – the DRESH study: straightforward study protocol for a randomized controlled trial. Trials..

[2] Lee KS (1998). Origin of chronic subdural haematoma and relation to traumatic subdural lesions. Brain Inj.

[3] de Araujo Silva DO (2012). Chronic subdural hematomas and the elderly: surgical results from a series of 125 cases: Old “horses” are not to be shot!. Surg Neurol Int..

[4] De Bonis P (2013). Antiplatelet/anticoagulant agents and chronic subdural hematoma in the elderly. PLoS One.

[5] Lee CH (2009). Spontaneous rapid reduction of a large acute subdural hematoma. J Korean Med Sci.

[6] Nakaguchi H, Tanishima T, Yoshimasu N (2001). Factors in the natural history of chronic subdural hematomas that influence their postoperative recurrence. J Neurosurg.

[7] Santarius T, Hutchinson PJ (2004). Chronic subdural haematoma: time to rationalize treatment?. Br J Neurosurg.

[8] Stippler M (2013). Chronic subdural hematoma patients aged 90 years and older. Neurol Res.

[9] Sun TF, Boet R, Poon WS (2005). Non-surgical primary treatment of chronic subdural haematoma: preliminary results of using dexamethasone. Br J Neurosurg.

[10] Delgado-Lopez PD (2009). Dexamethasone treatment in chronic subdural haematoma. Neurocirugia (Astur).

[11] Dran G (2007). Effectiveness of adjuvant corticosteroid therapy for chronic subdural hematoma: a retrospective study of 198 cases. Neurochirurgie.

[12] Berghauser Pont LM (2012). The role of corticosteroids in the management of chronic subdural hematoma: a systematic review. Eur J Neurol.

[13] Weigel R (2007). Angiotensin converting enzyme inhibition for arterial hypertension reduces the risk of recurrence in patients with chronic subdural hematoma possibly by an antiangiogenic mechanism. Neurosurgery.

[14] Wang D (2014). Effects of atorvastatin on chronic subdural hematoma: a preliminary report from three medical centers. J Neurol Sci.

[15] Hohenstein A (2005). Increased mRNA expression of VEGF within the hematoma and imbalance of angiopoietin-1 and -2 mRNA within the neomembranes of chronic subdural hematoma. J Neurotrauma.

[16] Nagy JA (2002). VEGF-A induces angiogenesis, arteriogenesis, lymphangiogenesis, and vascular malformations. Cold Spring Harb Symp Quant Biol..

[17] Wang D (2010). Membrane neovascularization and drainage of subdural hematoma in a rat model. J Neurotrauma.

[18] Wada T (2006). Local elevation of the anti-inflammatory interleukin-10 in the pathogenesis of chronic subdural hematoma. Neurosurg Rev.

[19] Frati A (2004). Inflammation markers and risk factors for recurrence in 35 patients with a posttraumatic chronic subdural hematoma: a prospective study. J Neurosurg.

[20] Stanisic M (2012). Chemokines as markers of local inflammation and angiogenesis in patients with chronic subdural hematoma: a prospective study. Acta Neurochir (Wien).

[21] Blum A (2014). HMG-CoA reductase inhibitors (statins), inflammation, and endothelial progenitor cells – New mechanistic insights of atherosclerosis. Biofactors.

[22] Sobrino T (2012). Increased levels of circulating endothelial progenitor cells in patients with ischaemic stroke treated with statins during acute phase. Eur J Neurol.

[23] Liu Y (2012). Beneficial effects of statins on endothelial progenitor cells. Am J Med Sci.

[24] Zhu JH (2004). Statins contribute to enhancement of the number and the function of endothelial progenitor cells from peripheral blood. Sheng Li Xue Bao.

[25] Dimmeler S (2001). HMG-CoA reductase inhibitors (statins) increase endothelial progenitor cells via the PI 3-kinase/Akt pathway. J Clin Invest.

[26] Araujo FA (2010). Atorvastatin inhibits inflammatory angiogenesis in mice through down regulation of VEGF, TNF-alpha and TGF-beta1. Biomed Pharmacother.

[27] Buttmann M (2007). Atorvastatin partially prevents an inflammatory barrier breakdown of cultured human brain endothelial cells at a pharmacologically relevant concentration. J Neurochem.

[28] Poulsen FR (2014). Perindopril and residual chronic subdural hematoma volumes six weeks after burr hole surgery: a randomized trial. Clin Neurol Neurosurg..

[29] Lennernas H (2003). Clinical pharmacokinetics of atorvastatin. Clin Pharmacokinet.

[30] Kapsalaki EZ (2007). Spontaneous resolution of acute cranial subdural hematomas. Clin Neurol Neurosurg.

[31] Fujimoto K (2014). Predictors of rapid spontaneous resolution of acute subdural hematoma. Clin Neurol Neurosurg..

[32] Marcikic M (2010). Spontaneous resolution of post-traumatic chronic subdural hematoma: case report. Acta Clin Croat.

[33] Miranda LB (2011). Chronic subdural hematoma in the elderly: not a benign disease. J Neurosurg.

[34] Labadie EL, Glover D (1976). Physiopathogenesis of subdural hematomas. Part 1: Histological and biochemical comparisons of subcutaneous hematoma in rats with subdural hematoma in man. J Neurosurg.

[35] Glover D, Labadie EL (1976). Physiopathogenesis of subdural hematomas. Part 2: Inhibition of growth of experimental hematomas with dexamethasone. J Neurosurg.

[36] Liu JN (2014). Attenuation of airway inflammation by simvastatin and the implications for asthma treatment: is the jury still out?. Exp Mol Med..

[37] Siddiqui AJ (2014). Rosuvastatin inhibits TIMP-2 and promotes myocardial angiogenesis. Pharmacology.

[38] Criner GJ (2014). Simvastatin for the prevention of exacerbations in moderate-to-severe COPD. N Engl J Med.

[39] National Heart, Lung and Blood Institute ARDS Clinical Trials Network (2014). Rosuvastatin for sepsis-associated acute respiratory distress syndrome. N Engl J Med.

[40] Barnes PJ (2014). Glucocorticoids. Chem Immunol Allergy..

[41] Brennan KM, Urschel KL (2014). Recovery of insulin sensitivity in mature horses after a 3 week course of dexamethasone therapy. Equine Vet J.

[42] Soininen K (2006). Muscle symptoms associated with statins: a series of twenty patients. Basic Clin Pharmacol Toxicol.

[43] Tikkanen MJ (2013). Effect of intensive lipid lowering with atorvastatin on cardiovascular outcomes in coronary heart disease patients with mild-to-moderate baseline elevations in alanine aminotransferase levels. Int J Cardiol.

[44] Kalantari S, Naghipour M (2014). Statin therapy and hepatotoxicity: appraisal of the safety profile of atorvastatin in hyperlipidemic patients. Adv Biomed Res..

