# Supplementary material for: Effect of ATorvastatin On Chronic subdural Hematoma (ATOCH): a study protocol for a randomized controlled trial
Source: Trials. 2015 Nov 18;16:528. doi: 10.1186/s13063-015-1045-y (PMC4652431; doi:10.1186/s13063-015-1045-y)
Supplement: Additional file 1: — Clinical Trial Certificate of hospital centers participating in the trial. Medical centers in China participating in the study, ethics committee and the certificate number. (PDF 48 kb) [file 13063_2015_1045_MOESM1_ESM.pdf]

Medical Centers in China Participating in the Study

Medical Centers	Ethics Committee	Certificate No.
1.Tianjin Medical University, General Hospital	Medical Ethics Committee of Tianjin medical university general hospital	IRB2012-028-02
2.Peking Union Medical College Hospital	Ethics Committee of Peking Union Medical College Hospital Affiliated to Chinese Academy of Medical Sciences	B-107
3.Prince of Wales Hospital, Hong Kong	Pharmacy and Poisons Board of Hong Kong	PR/CT0257/2014(SC)
4.Qilu Hospital of Shandong University	Medical Ethics Committee of Qilu Hospital Affiliated to Shandong University	No.2013049
5.The first affiliated hospital of zhengzhou university	Clinical trial ethics committee of the first affiliated hospital of zhengzhou university	MED-2014-09
6.The Second Affiliated Hospital of Zhejiang University School of Medicine	Human research ethics committee of the Second Affiliated Hospital of Zhejiang University School of Medicine	STU-2014-104
7.First affiliated hospital of fujian medical university	Medical Ethics Committee of First affiliated hospital of fujian medical university	[2014]053
8.First affiliated hospital of shanxi medical university	Science / Medical experimental ethics committee of First affiliated hospital of shanxi medical university	BHR-I00-301
9.General hospital of Ningxia medical university	Scientific experiment ethics committee of Ningxia medical university General hospital	No.20140709
10.Linyi People’s Hospital	Medical Ethics Committee of Linyi People’s Hospital	No.2014047
11.Southwest Hospital The First Affiliated Hospital of the Third Military Medical University	Ethics Committee of the First Affiliated Hospital of Third Military Medical University, PLA	No.2014-20
12.Tangdu Hospital, The Second Affiliated hospital of the Forth Military Medical University	IEC of Institution for National Drug Clinical Trials, Tangdu Hospital, Forth Military Medical University	No.2014064
13.The affiliated hospital of Xuzhou medical college	Medical Ethics Committee of affiliated hospital of Xuzhou medical college	XYFY2014025
14.The First Affiliated hospital of Harbin Medical University	Medical Ethics Committee of First hospital Affiliated to Harbin Medical University	No.201336
15.Xijing Hospital，The First Affiliated hospital of the Forth Military Medical University	IEC of First Affiliated hospital of Forth Military Medical University	KY20140813
16.Inner Mongolia people's hospital	Ethics Committee of Inner Mongolia people's hospital	B-20140627
