# Supplementary material for: Effect of ATorvastatin On Chronic subdural Hematoma (ATOCH): a study protocol for a randomized controlled trial
Source: Trials. 2015 Nov 18;16:528. doi: 10.1186/s13063-015-1045-y (PMC4652431; doi:10.1186/s13063-015-1045-y)
Supplement: Additional file 2: — MGS-GCS. Patients were evaluated using the Glasgow Coma Scale and Markwalder’s Grading Scale. Only patients with Grade 0–2 chronic subdural hematoma (CSDH) were selected for atorvastatin treatment in this study. (PDF 34 kb) [file 13063_2015_1045_MOESM2_ESM.pdf]

MGS-GCS

Patients were evaluated using the Glasgow Coma Scale and Markwalder's Grading Scale

Patient's Grade	Glasgow Coma Scale	Markwalder's Grading Scale
Grade 0	Glasgow Coma Scale score of 15	Normal neurological status without any symptoms
Grade 1	Glasgow Coma Scale score of 15	Without neurological deficits, but with symptoms such as headache or unsteady gait
Grade 2	Glasgow Coma Scale score of 13 to 14	Focal neurological deficits, such as drowsiness or disorientation, or variable neurological deficits, such as hemiparesis
Grade 3	Glasgow Coma Scale score of 9 to 12	With stupor but appropriate responses to noxious stimuli and several focal neurological signs such as hemiplegia
Grade 4	Glasgow Coma Scale score of less than 9	Coma with absent motor responses to noxious stimuli and decerebrate or decorticate posturing

Only patients with Grade 0-2 CSDH were selected for atorvastatin treatment in this study.

References：

1. Markwalder TM, Steinsiepe KF, Rohner M, Reichenbach W, Markwalder H. The course of chronic subdural hematomas after burr-hole craniostomy and closed-system drainage. Journal of neurosurgery. Sep 1981;55(3):390-396.
2. Sun TF, Boet R, Poon WS. Non-surgical primary treatment of chronic subdural haematoma: Preliminary results of using dexamethasone. British journal of neurosurgery. Aug 2005;19(4):327-333.
